# Vaccine Passports and Political Legitimacy: A Public Reason Framework for Policymakers

**DOI:** 10.1007/s10677-022-10361-1

**Published:** 2023-02-15

**Authors:** Anne Barnhill, Matteo Bonotti, Daniel Susser

**Affiliations:** 1 Johns Hopkins Berman Institute of Bioethics, Johns Hopkins University, Deering Hall, 1809 Ashland Avenue, Baltimore, MD 21205 USA; 2 Politics and International Relations, School of Social Sciences, Faculty of Arts, Monash University, Clayton, VIC 3800 Australia; 3 College of Information Sciences & Technology, Rock Ethics Institute, The Pennsylvania State University, E325 Westgate Building, University Park, PA 16802 USA

**Keywords:** Vaccine passports, COVID-19, Political legitimacy, Public reason

## Abstract

As the COVID-19 pandemic continues to evolve, taking its toll on people’s lives around the world, vaccine passports remain a contentious topic of debate in most liberal democracies. While a small literature on vaccine passports has sprung up over the past few years that considers their ethical pros and cons, in this paper we focus on the question of when vaccine passports are politically legitimate. Specifically, we put forward a ‘public reason ethics framework’ for resolving ethical disputes and use the case of vaccine passports to demonstrate how it works. The framework walks users through a structured analysis of a vaccine passport proposal to determine whether the proposal can be publicly justified and is therefore legitimate. Use of this framework may also help policymakers to design more effective vaccine passports, by incorporating structured input from the public, and thereby better taking the public’s interests and values into account. In short, a public reason ethics framework is meant to encourage better, more legitimate decision-making, resulting in policies that are ethically justifiable, legitimate and effective.

## Introduction

As the COVID-19 pandemic continues to evolve, taking its toll on people’s lives around the world, vaccine passports remain a contentious topic of debate in most liberal democracies (Drew 2022). This should come as no surprise: vaccine passports raise a number of ethical issues about which people can reasonably disagree. On the one hand, vaccine passports can help to promote (and restore) greater freedom of movement, economic growth, and access to key job and educational opportunities without threatening public health. On the other hand, they might result in various forms of unequal treatment and discrimination, compound existing structural inequalities and injustices, and infringe upon people’s privacy. They could even *undermine* public health, if they encourage riskier behaviours based on overconfidence. A small literature on vaccine passports has sprung up over the past few years that considers these ethical pros and cons (Ada Lovelace Institute 2021; Brown et al. 2021; Giubilini and Kennedy 2021; Hall and Studdert 2020; Kofler and Baylis 2020; Nuffield Council on Bioethics 2020; Persad and Emanuel 2020; Voo et al. 2020). Less discussed, however, are questions concerning the *political legitimacy* of vaccine passports and, more generally, of policy responses to COVID-19 (Bernstein et al. 2021). There are distinct characterizations of legitimacy in the literature (Peter 2017); the one at issue here is the view that a law or policy is legitimate when the political entity that passes and/or enforces it has the right to do so (Buchanan 2002).

Policy responses to COVID-19 involve high-stakes decisions, requiring trade-offs between different values—policies may prevent some harms but cause others, limit liberty, and reshape the public’s expectations of government. There is disagreement among both policymakers and the public about how to navigate these decisions. And this confluence of high stakes and significant, reasonable disagreement raises pressing questions about what it takes for COVID-19 policies (and, more generally, public health policies) to be legitimate. Should policymakers defer to experts when making policy decisions? Should they do what a majority of the public prefers? Should they follow their own moral compasses, doing what they believe is the most ethical course of action? Or something else? How we answer these questions should be informed by a view of political legitimacy—that is, a theory of the conditions under which governments have the right to implement specific laws and policies to tackle COVID-19.

In this paper we endorse a specific account of the source of political legitimacy, according to which a government’s right to pass and/or enforce laws and policies is grounded in the idea of public reason (Rawls 2005), i.e., the view that political rules ought to be justified to those subject to them by reasons they can accept (at some level of idealization). Based on this view, we put forward a ‘public reason ethics framework’ for resolving disputes concerning vaccine passports and demonstrate how it works. The framework walks users through a structured analysis of a vaccine passport proposal to determine whether the proposal can be publicly justified (and is therefore, as we explain in what follows, legitimate). Use of this ethics framework may advance three aims: helping policymakers to design legitimate vaccine passports; helping them to design more effective vaccine passports; and, if the framework is used to solicit structured input from the public, rendering policymakers and policymaking more accountable to the public. In short, a public reason ethics framework is meant to encourage better, more legitimate decision-making, resulting in policies that are ethically justifiable, legitimate and effective.

Our analysis proceeds as follows. First, we provide a brief overview of the recent debates concerning the practical and ethical issues concerning COVID-19 vaccine passports. Second, we introduce our ethics framework, noting its links with prior ethics frameworks for public health policies. Third, we outline the idea of public reason central to the conception of political legitimacy that we endorse in the paper. We focus specifically on an *accessibility conception of public reason*, explaining how it differs from, and is preferable to two competing approaches, intelligibility and shareability conceptions. Finally, we combine our insights to fully develop our ethics framework, explaining how it can help policymakers to develop a politically legitimate vaccine passport system.

## Vaccine Passports: Scientific Evidence and Ethical Issues

From the moment it became clear that managing the COVID-19 pandemic would require massively curtailing freedom of movement—shutting down or limiting access to factories, offices, restaurants, and other public places for significant portions of the population—a search began for ways to safely let people move around again. Among the many tools proposed and developed, vaccine passports (also sometimes called ‘immunity passports’ or ‘immunity certificates’)^1^ have been one of the most controversial.

The basic idea is intuitive: to mitigate the spread of a contagious disease, not everyone needs to quarantine—if individuals can be identified who are unlikely to become infected and transmit the disease, either because they are vaccinated or have already recovered from infection and developed natural immunity, then a document or ‘passport’ certifying such status should enable them to move freely without threatening public health (Hall and Studdert 2021).

Yet proposals for vaccine passports have met with considerable opposition. In the early stages of the pandemic, while the virus was still something of a mystery and prior to the development of effective COVID-19 vaccines, experts raised a number of concerns about the practical feasibility of immunity passports. While there was reason to expect that surviving infection conferred some immunity against reinfection, the *degree* and *duration* of immunity were unclear (Kofler and Baylis 2020). Serological tests used to measure the level of antibodies in an individual’s blood were unreliable (Phelan 2020) and in short supply (Brown et al. 2020), and there was disagreement about the optimal timing of tests (Voo et al. 2020). Questions were raised about who exactly—which institutional actors—could and should verify immunity (Salmon et al. 2022), and how to avoid the inevitable rise of fraudulent certificates (Hall and Studdert 2020). And some worried that privileging people who had survived infection, by granting them freedom of movement denied to others, would incentivize people to intentionally seek out infection, thus furthering the spread of the virus rather than helping to contain it (Phelan 2020).

Even with the introduction of COVID-19 vaccines, some of these practical considerations remain unresolved. Of course, the widespread availability of vaccines has altered the incentives vaccine passports produce—rather than inadvertently encouraging people to seek out infection, the freedom of movement vaccine passports allow should (and often does) encourage vaccination. Yet vaccination and previous infection remain imperfect proxies for likelihood of future infection or transmission, and the emergence of new viral variants, which produce new and different immune responses, means that the science will likely remain unsettled (Ada Lovelace Institute 2021). The vaccines on the market (and most of those under development) are designed to make people less sick when they are exposed to the virus (Stokel-Walker 2022) but do not seem to prevent transmission (Franco-Paredes 2022), and there has been less focus on developing vaccines designed to stop people from becoming infected in the first instance.^2^ Indeed, the Omicron and BA2 variants of COVID-19 have made it clear that protection against infection wanes over time, allowing the virus to circulate even among the vaccinated (Andeweg et al. 2022). Furthermore, immunity from illness seems limited (Dolgin 2021)—many people have had COVID-19 multiple times (in some cases, with as little as six weeks between illnesses). Moreover, the evidence of efficacy of second boosters is relatively disappointing, as one Israeli study showed (Bar-On et al. 2022). And uncertainty remains regarding the reliability of testing (Soni et al. 2022).

Another problem is that by framing risk of infection in binary terms—having a vaccine passport means one is safe, lacking a passport means one is not—some worry that vaccine passport regimes could distort the public’s understanding of the real risks COVID-19 poses and undermine other public health strategies, such as masking and social distancing (Ada Lovelace Institute 2021). Indeed, vaccination and previous infection may be providing false assurance, leading many people to feel comfortable taking off their masks despite the risks this entails for them and for others (World Health Organization 2021). Another common concern is that foisting vaccine passports on people could frustrate their sense of autonomy, ultimately increasing mistrust in health institutions and leading to more people refusing to get vaccinated (Ada Lovelace Institute 2021). Moreover, the introduction of vaccine passports has triggered intense resistance in a number of jurisdictions, with protests against vaccination mandates providing a gateway to more extreme groups, such as QAnon and the Sovereign Citizen movement (Roose 2021), with attendant political destabilisation.

Beyond concerns about practical feasibility, effectiveness, and destabilizing effects, experts also point to a number of ethical concerns, some taking issue with the idea of vaccine passports in general and others targeting the specific technologies that would facilitate them. For a start, there are worries about equal treatment. At one level, the very purpose of vaccine passports is to facilitate discrimination, allowing those who are immunized against COVID-19 to move freely while limiting the movement of those who are not. This in itself is not a problem, at least on the surface, because the discrimination is not unjustifiable—it tracks a legitimate distinction that serves a significant social end (Hall and Studdert 2020; Persad and Emanuel 2020). Yet some worry that if there are principled reasons for not getting vaccinated—motivated, for example, by religious objections—then it would be wrong to ‘penalize’ people for refusing vaccination (Hall and Studdert 2021). Worse, if the distribution of immunity across the population (and the ability to prove it) is, in part, a function of existing structural inequalities, with already marginalized groups lacking equal access to vaccines and testing, then conditioning freedom of movement on immunity is likely to perpetuate prior injustices (Brown et al. 2021; Hall and Studdert 2020; Voo et al. 2020). Furthermore, this could create *new* forms of injustice if those lacking immunity are stigmatized for it (Hall and Studdert 2020) or if they are deprived of access to important goods, such as employment or insurance (Kofler and Baylis 2020).

Furthermore, there are worries about privacy. In general, as Kofler and Baylis (2020, p. 380) argue, ‘[m]onitoring erodes privacy’, and implementing an effective vaccine passport program necessarily involves significant monitoring. It requires tracking who is and is not vaccinated, creating records of COVID-19 testing, and—more broadly—developing systems that could facilitate ‘troublesome monitoring of people’s movements and health statuses’ (Brown et al. 2021). Specifically, ethicists raise three privacy-related concerns. First, some worry that widespread use of vaccine passports could create unwarranted pressure on individuals to get tested or vaccinated and to disclose their health status to others—especially if vaccine passports became conditions of employment (Brown et al. 2020; Nuffield Council on Bioethics 2020). Second—and relatedly—the decision to *not* disclose one’s health or vaccination status could become a source of suspicion or stigma (Nuffield Council on Bioethics 2020). And third, the technologies used to implement vaccine passports at scale could open the door to more invasive and lasting forms of surveillance (Ada Lovelace Institute 2021; Giubilini and Kennedy 2021).

This last concern, about what some call ‘surveillance creep’ (i.e., the use of surveillance technologies designed for one purpose to facilitate another) is especially acute when considering *digital* vaccine passports. New technologies tend to outlive their originally intended uses, so it is important when developing them to consider other ways they could be utilized—to consider ‘what kind of future vaccine passports would take us towards’ (Giubilini and Kennedy 2021). The Electronic Frontier Foundation, a US digital civil rights group, worries that digital vaccine passports could normalize requiring ‘proof-of-status’ documents as conditions of entry into public spaces (Hancock and Gullo 2020). They and others worry that these requirements could expand from vaccination status to the disclosure of other private health information, such as HIV status or genetic information (Kofler and Baylis 2020). Digitizing the personal health information necessary for vaccine passports could result in that information flowing to unexpected third parties, such as law enforcement agencies or insurance companies (Ada Lovelace Institute 2021; Kofler and Baylis 2020); it could expose that information to potential data breaches (Hancock and Gullo 2020); and, because many digital vaccine passport programs rely on private technology firms for their underlying infrastructure, some worry such programs could increase the technology industry’s already significant power over people’s lives (Giubilini and Kennedy 2021).

The question, then, is how to balance these concerns against the considerable benefits vaccine passports promise—greater freedom of movement; the economic benefits of allowing people to safely shop, dine in restaurants, visit cultural institutions and, more broadly, to re-enter schools, universities, and the workforce; incentivising higher vaccination rates and thus reducing the burden on the health system; and the sense of security people would feel knowing that those around them are unlikely to transmit COVID-19.^3^ Moreover, how should policymakers manage inevitable disagreements about what the proper balance is?

## Towards an Ethics Framework for Vaccine Passports

In view of the concerns outlined in the previous section, how should a legitimate vaccine passport policy be devised? In what follows, we offer one possible approach: policymakers can use an ethics framework, which is a practical instrument for analyzing the justifiability of a potential policy. We offer an ethics framework that walks users through a structured analysis of a policy by posing questions that incorporate ethical and other considerations in a systematic way. This framework is a variant of the framework developed by Barnhill and Bonotti (2022) to analyse a different area of public health policy, i.e., healthy eating policy. That framework draws, in turn, on prior ethics frameworks for public health policies, in particular those developed by Childress et al. (2002), Kass (2001), and ten Have et al. (2013). These ethics frameworks are designed for end-users—such as public health practitioners or members of the public—who may have little understanding of ethical theory or political legitimacy, and may have no idea how to apply an ethical theory or theory of legitimacy to a specific policy context. Ethics frameworks are practical tools designed to lower barriers of entry into analyzing the ethics and legitimacy of policies.

Core to ethics frameworks for public health is a recognition that public health measures implicate a range of important values, that there can be trade-offs or tensions between values (for example, a public health policy may improve health but threaten privacy), and that the ethical analysis of proposed public health measures requires identifying these trade-offs and assessing whether they are justified (for example, by asking whether the threat to privacy is justifiable in light of the likely public health benefits). Given that people understand and weigh the values central to public health in different ways, we should expect disagreement about the justifiability of specific public health measures; some ethics frameworks take this into account. For example, according to Kass (2001, p. 1781), decisions should be made not based on the majority’s will but via fair procedures and deliberative processes that also take into account the interests and views of the minority. Kass’s emphasis on giving minority voices their due points to the importance of public justification, an aspect also highlighted by Childress et al. (2002, p. 173).

We agree that public justification should be central to public health decision-making. Public health policies and interventions, including the introduction of vaccine passports, ought not to be implemented from on high by policymakers without taking into account citizens’ views—especially when such policies risk infringing upon persons’ core rights and liberties. But neither should those decisions be driven solely by majority rule. One problem with grounding the legitimacy of laws and policies in majority rule alone (e.g., Dahl 1956; May 1952) is that the latter may produce policies based, for example, on unreflective views rooted in misinformation, racist attitudes, or controversial religious beliefs. These kinds of views may in different ways undermine the public justifiability and legitimacy of public health policies and interventions. Furthermore, pure majoritarianism may neglect key ethical considerations, not least the rights and interests of minority groups.

Likewise, we believe that an approach centred on public justification as a source of political legitimacy is also preferable to one that grounds legitimacy in utilitarianism (e.g., Binmore 2000). Utilitarianism demands that policies and laws maximize citizens’ happiness. This is an ethical perspective that received support from prominent scholars during the pandemic (e.g., Savulescu et al. 2020). However, it is also an approach that has been significantly criticized for its failure to assign sufficient importance to individual rights and the impact of COVID-19 policies on members of vulnerable groups (Herron and Manuel 2022; Weismann and Holder 2021). To use Rawls’s famous words, ‘[utilitarianism] is the consequence of extending to society the principle of choice for one man, and then, to make this extension work, conflating all persons into one…Utilitarianism does not take seriously the distinction between persons’ (1971, p. 27).

Our view is therefore that public health interventions should be developed through a process that takes citizens’ views into account when designing and justifying policies *and also* applies standards about which views are allowed into this process. In other words, a legitimate decision-making process for public health policies involves accountability to citizens and responsiveness to their views while rejecting certain views as inappropriate for the task of designing and justifying legitimate public policies. In what follows, we explain this view of legitimacy, which is a version of what is known as an *accessibility conception of public reason*. We start by explaining what public reason involves.

## Public Reason and Vaccine Passports

The idea of public reason is central to contemporary liberal political philosophy, and in particular to political liberalism (Gaus 2010; Quong 2011; Rawls 2005). It is the idea that to be legitimate, political rules ought to be justified to those individuals who are subject to them by reasons they can accept (at some level of idealization).

The idea of public reason is grounded in two fundamental premises. First is the acknowledgment that contemporary liberal democracies are deeply diverse—i.e., that they are characterized by the fact of reasonable disagreement (or reasonable pluralism) (Rawls 2005). Such disagreement is ‘reasonable’ in the sense that it occurs among persons who are ‘ready to propose principles and standards as fair terms of cooperation and to abide by them willingly, given the assurance that others will likewise do so’ (Rawls 2005, p. 49), and who recognize the ‘burdens of judgment’, i.e., ‘the many hazards involved in the correct (and conscientious) exercise of our powers of reason and judgment in the ordinary course of political life’ (Rawls 2005, p. 56). The burdens of judgment include, for instance, complex empirical evidence, vague concepts, and different ways of weighing conflicting considerations when evaluating empirical and moral arguments.

Second, the idea of public reason is premised on the notion of citizens as free and equal persons, a core principle of political liberalism. According to this view, ‘[e]ach of us is free in the sense of not being naturally subject to any other person’s moral or political authority, and we are equally situated with respect to this freedom from the natural authority of others’ (Quong 2022). If citizens are compelled to obey political rules that are not grounded in reasons they could accept at some level of idealization, that is tantamount to denying their free and equal status.

A key feature of public reason, and one that has received particular attention in the contemporary literature, is the exclusion of certain reasons from the process of public justification. Since public reason requires that political rules be justified by appealing to reasons that all citizens could accept at some level of idealization, reasons that do not fit this criterion are unsuitable for public justification, i.e., they are ‘non-public’ reasons. Such reasons include not only those based on views that fail to recognize the rights and liberties of others but also those grounded in religious, ethical, and philosophical doctrines about which citizens can reasonably disagree. For example, a public health intervention that is justified by appealing to reasons grounded in Catholic doctrine would probably be considered illegitimate by non-Catholics. But not all political liberals believe that religious and other controversial non-public reasons are unsuitable for public justification, as we explain next.

### Intelligible Reasons and Vaccine Passports

Defenders of the so-called ‘intelligibility’ or ‘convergence’ view of public reason argue that political rules are legitimate if they are justified to different people based on their different ‘intelligible’ reasons (Gaus 2010; Vallier 2014). In this view, ‘A’s reason R_A_ is intelligible for members of the public if and only if members of the public regard R_A_ as epistemically justified *for A* according to *A’s evaluative standards*’ (Vallier 2014, p. 106, emphasis added), where evaluative standards should be understood as ‘a set of prescriptive and descriptive standards or beliefs that a member of the public takes to justify her reason affirmations and that enables her to order her moral and political proposals’ (Vallier 2016, p. 602).

Thus, under the intelligibility conception a political rule (e.g., a public health intervention) may be justified to some citizens based on reasons grounded in Catholicism and to other citizens based on reasons grounded in Islam (or, for that matter, in Kantian or Millian liberalism, and so on). This conception of public reason has been extensively criticized in the literature. For example, it has been argued that it is grounded in a controversial relativist conception of justification (Quong 2011, pp. 261–273); that it fails to provide assurance among members of a liberal society (Macedo 2010); and that it allows most political rules to be defeated by merely intelligible reasons (Eberle 2011, pp. 300–1). We accept (without further argument) Quong and Macedo’s points but would like to zoom in on Eberle’s criticism, especially since the link between intelligibility and state inaction has also been acknowledged by one of intelligibility’s key proponents, Kevin Vallier (2016, p. 603; 2019, p. 115; see also Barnhill and Bonotti 2022, Ch. 5).^4^

The problem highlighted by the state inaction critique is that any intelligible reason (including reasons grounded in controversial religious or philosophical beliefs) can potentially defeat a policy such as a public health intervention. In the specific case of vaccine passports, because many citizens will likely object to them based on non-public reasons—e.g., objections to vaccine passports by UK clergy, on the basis of ‘serious issues of conscience related to the ethics of vaccine manufacture or testing’ (Sherwood 2021)—it seems implausible that intelligibility will allow any legislation at all in this area.

At this point, though, a critic might point out that intelligibility does not necessarily rule out vaccine passports. For example, Gaus (2010, p. 362)—another key proponent of the intelligibility conception—defends the idea of a ‘common good requirement’, according to which.

a moral rule that can be endorsed by all free moral agents must be consistent with the common good of all, at least in the minimal sense of not itself posing a threat to anyone’s good…Free and rational moral agents must consider how their overall systems of values, goals, and ends will fare under a proposed moral rule, and so they cannot have good reason to embrace a moral rule that poses a threat to what they care for. This common good requirement not only includes freedom to act on one’s aims, goals, and purposes but also protection from basic harms. There cannot, I think, be any doubt that a principle prohibiting basic harms (a strong yet defeasible principle) is fully justified. Rules against harms are part of all moral systems and are among the rules that children first recognize as necessary to social life. In pluralistic cultures with strong commitment to the perspective of agency, harm rules are apt to focus on protection of the basic goods for effective agency.

Following Gaus, one might argue that since being exposed to high risk of debilitating illness or disease (such as COVID-19) is a basic harm, a policy that prevents this harm (i.e., vaccine passports) is demanded by the ‘common good requirement’, and otherwise intelligible reasons against vaccine passports cannot act as defeaters for this policy. However, this conclusion is grounded in the assumption that individuals do not have any alternative option to protect themselves from the virus. This assumption seems to be unfounded since individuals who would like to protect themselves from the virus do have an alternative option, i.e., *they* can get vaccinated (Savulescu and Giubilini 2020). Given vaccines’ high effectiveness in reducing debilitating COVID-19 illness or disease, it seems that this would be sufficient to prevent the kind of basic harm that Gaus’s ‘common good requirement’ aims to rule out. Therefore, while Gaus’s common good requirement may justify mandatory vaccination,^5^ it does not justify mandatory vaccine passports.

### Shareable Reasons and Vaccine Passports

Instead of a convergence or intelligibility conception, most political liberals endorse a ‘consensus’ account of public reason. The most straightforward version of this account states that public reasons should be *shared* among members of the public (e.g., Bohman and Richardson 2009; Schwartzman 2011, p. 378), where ‘A’s reason R_A_ is shared with the public if and only if members of the public regard *R*_*A*_ as epistemically justified *for each member of the public*, including A’ (Vallier 2014, p. 110, emphasis added).

The shareability conception of public reason, however, faces two serious objections, both of them leading to the conclusion that, like intelligibility, it can result in state inaction. Consider, first, Vallier’s point that shareability allows a reason to justify political rules only if ‘each citizen will affirm the reason *as her own* at the right level of idealisation’ (Vallier 2014, p. 109, original emphasis). For that to be possible, the reason must be compatible with a person’s ‘*subjective motivational set*’ (Vallier 2011, p. 370, original emphasis), i.e., with their desires, beliefs, ‘dispositions of evaluation, patterns of emotional reaction, personal loyalties, and various projects’ (Williams 1981, p. 105, cited in Vallier 2011, p. 387, note 11). If a person’s motivational set includes, say, the belief that vaccine passports (and/or vaccines more generally) are in tension with their religious or ethical convictions, then it may be difficult for them to ‘share’ a reason in support of vaccine passports. Even if that reason appeals, say, to the common good, or to the advancement of rights and liberties that they would in principle not object to when appealed to in order to justify other policies, when that reason conflicts with their subjective motivational set it may lose its justificatory strength. Therefore, like intelligibility, shareability is also likely to limit the range of policies (including the range of public health interventions) that are publicly justified. And it is unlikely that vaccine passports will be among them.

Yet this criticism is not entirely persuasive. Unlike Vallier, consensus theorists of public reason often add a key condition to the shareability conception of public reason, i.e., the view that reasons must be shareable among *reasonable* persons, i.e., persons who (as we already explained earlier) ‘are ready to propose principles and standards as fair terms of cooperation and to abide by them willingly, given the assurance that others will likewise do so’ (Rawls 2005, p. 49)—a condition that we endorse. Since being reasonable involves having a prior commitment to liberalism, reasonable persons give special priority to distinctly public reasons in their conception of justification (Quong 2011; Watson and Hartley 2018). This view is captured by Rawls’s famous statement that when our political and comprehensive views are in conflict, ‘[the political conception’s] values normally outweigh whatever other values oppose them, at least under the reasonably favorable conditions that make a constitutional democracy possible’ (2005, p. 155). This has two important implications. First, it explains why—unlike under the intelligibility conception—individual morally/religiously-driven defeaters are not weighty enough to be given their due in public reason. Second, it clarifies why the state inaction criticism does not hold: if public justification is addressed to reasonable persons, and such persons can in fact integrate the pertinent public reasons into their motivational set, then shared public reasons do not lose their justificatory strength.^6^

But the shareability conception of public reason faces a second, more serious challenge: it neglects, or at least seriously underestimates, the reasonable pluralism that characterizes contemporary liberal societies. As Vallier points out,

[i]f public reason liberals adopt shareability, and they want to avoid a particularly extreme libertarian version of public reason, then they must argue that the number of shared reasons among members of liberal societies is quite large. But this is tantamount to denying reasonable pluralism, something that all public reason liberals must accept. Public reason liberalism is motivated by the idea that reasonable people will inevitably disagree about many of the most important questions in life; accordingly, public reason liberals cannot adopt a requirement on public reasons that ignores this fact. For this reason, public reason liberals should reject shareability requirements (2011, pp. 387-8).

Since, as well as proposing fair terms of cooperation, reasonable persons must also acknowledge the burdens of judgment (Rawls 2005, p. 54), and since such burdens imply that there will be very few (if any) shared reasons among reasonable members of the public—even if epistemically and normatively idealized (Badano and Bonotti 2020, pp. 43–44)—then shareability seems to impose unreasonable burdens of justifications upon reasonable members of a liberal society. Furthermore, and as a result, like intelligibility it will also result in state inaction (see Barnhill and Bonotti 2022, Ch. 5).

It is our position that because both the intelligibility and the shareability conceptions of public reason result in state inaction—as regards vaccine passports but also, plausibly, other areas of public health—they should be rejected. Our point, in rejecting the intelligibility and shareability conceptions, is not to beg the question of whether vaccine passports are politically legitimate or not, by simply assuming that they are and that, therefore, the intelligibility and shareability conceptions of public reason are wrong. After all, the purpose of an ethics framework like the one that we develop in this paper is precisely to help policymakers to decide what action to take and one possible action is, of course, rejecting vaccine passports altogether. However, under realistic social conditions, and especially given the fact of reasonable pluralism, the intelligibility and shareability conceptions de facto rule out vaccine passports as a politically legitimate policy option, thus precluding any type of debate on this matter among policymakers. This seems unsatisfactory if one wants to remain open to the possibility that vaccine passports (and other public health measures) may in principle be legitimate. That possibility—and, therefore, any scope for public debate among policymakers as well as members of the public—remains real only if one endorses a different conception of public reason. But what does this alternative conception look like?

### Accessible Reasons and Vaccine Passports

The ‘accessibility’ conception of public reason—endorsed, for example, by Audi (2011) and by Rawls (2005) himself—navigates a third way between intelligibility and shareability (Badano and Bonotti 2020). Like shareability, it is a consensus conception of public reason but one which takes more seriously the fact of reasonable pluralism that characterizes contemporary liberal societies. Instead of demanding that reasons for political rules be shared among all reasonable members of the public, it requires that those reasons be *accessible* to them at the right level of idealization, i.e., that citizens ‘regard [a reason] R_A_ as epistemically justified *for A* according to *common evaluative standards*’ (Vallier 2014, p. 108, emphasis added). Accessibility occupies a middle ground between intelligibility and shareability because, on the one hand, it limits the range of reasons allowed into the process of public justification—reasons that are based on non-shared evaluative standards (e.g., the evaluative standards that are central to Islam or Catholicism) would not pass the accessibility test—while, on the other hand, it does not demand that all citizens share a reason in order for that reason to be suitable for public justification (Badano and Bonotti 2020). To understand why, we need to unpack the concept of ‘evaluative standards’. We draw on Barnhill and Bonotti's (2022) analysis and identify two main categories of such standards: moral standards and epistemic/factual standards.

#### Moral Evaluative Standards and Vaccine Passports

The most common category of evaluative standards discussed in the public reason literature includes *moral* evaluative standards. Shared moral evaluative standards include political values that are widely endorsed in liberal democratic societies, such as basic rights and liberties, equality of opportunity and the common good (Rawls 2005, pp. 223–4). Basic rights and liberties include ‘freedom of thought and liberty of conscience; the political liberties and freedom of association, as well as the freedoms specified by the liberty and integrity of the person; and…the rights and liberties covered by the rule of law’ (Rawls 2005, p. 291). By contrast, non-shared moral evaluative standards are those stemming from specific religious, ethical and philosophical traditions, about which people reasonably disagree.

Under accessibility, when a person appeals to shared moral evaluative standards in order to justify a policy such as vaccine passports, other people may find their reason accessible, and therefore suitable for public justification, *even if they do not share that reason.* To understand why, consider this example provided by Jonathan Quong (2011, p. 205), whose consensus conception of public reason also seems to be an instance of accessibility.

Two friends, Tony and Sara, are debating whether the Catholic Church should be legally compelled to hire female priests. Tony appeals to the value of religious liberty to justify the Church’s right to only employ male priests, while Sara appeals to the values of gender equality and non-discrimination to justify the view that the Church should be obligated to hire female priests. Since both religious liberty, on the one hand, and gender equality and non-discrimination, on the other hand, are widely shared moral values in liberal democratic societies, and assuming that Tony and Sara are committed to both sets of values (despite the fact that they may assign different weight to them in their reasoning), they are both providing accessible reasons for the policies they defend—even though they do not *share* a reason for or against the policy at stake. In this sense, an accessible reason is one that provides a ‘plausible [or reasonable] balance of political values…[i.e., it must recognize] that there are multiple political values at stake, and [offer] a plausible explanation as to why one public value ought to be prioritized over the other in cases of this kind’ (Quong 2011, p. 209; see also Rawls 2005, p. 243).

When it comes to vaccine passports, those in favour might appeal to the fact that they would improve public health outcomes and save lives (thus also enabling individuals to fully enjoy their basic rights and liberties), or that they would help restart economic activity, which would—in a different way—also contribute to the common good. Conversely, those who oppose vaccine passports might appeal to the value of privacy—another important shared value in contemporary liberal democracies—or to the fact that their use may impose disproportionate ‘strains of commitment’ (Rawls 1971, p. 153; Quong 2006) on already vulnerable and marginalized groups, for example if, as we have already pointed out, the distribution of immunity across the population (and the ability to prove it) is, in part, a function of existing structural inequalities. On both sides of the debate, therefore, we find an appeal to widely shared political values. As long as those on each side of the debate give serious consideration to the values prioritized by the other, and they explain why (a) different value(s) should be prioritized over (an)other(s), their position will be publicly justified and, according to the accessibility conception of public reason, legitimate.

This last point is important: recall that in the case of intelligibility, any intelligible reason against a policy can defeat it. This is not the case with accessibility. As a consensus view of public reason, accessibility presupposes that reasonable persons—who form the constituency of public reason—always prioritize public reasons over their comprehensive doctrines. Therefore, if a policy is publicly justified based on an accessible public reason, it is still legitimate for all reasonable members of the public, including those who may oppose it based on a different accessible public reason (e.g., one grounded in a different balance of shared political values). Accessibility therefore differs from intelligibility by not providing a pathway for any objection that satisfies the public reason standard (i.e., any intelligible reason or accessible reason) to defeat political rules. Accessibility can also help us to exclude non-shared moral evaluative standards from public justification. These may include the moral evaluative standards associated with religious, ethical and philosophical traditions that are controversial in contemporary liberal democratic societies. Hence, for example, those who support or oppose vaccine passports based on moral arguments contained in some religious text, when those arguments are not widely shared, are providing inaccessible reasons for their position.

#### Epistemic/Factual Evaluative Standards and Vaccine Passports

A second type of evaluative standard, equally important but less widely discussed in the public reason literature, includes epistemic and factual evaluative standards. According to Rawls, for example, public reason includes ‘the methods and conclusions of science when these are not controversial’ (Rawls 2005, p. 224) as well as ‘plain truths now widely accepted, or available, to citizens generally’ (Rawls 2005, p. 224) and ‘presently accepted general beliefs and forms of reasoning found in common sense’ (Rawls 2005, p. 224). In other words, for a policy to be publicly justified, it must not only be grounded in a reasonable balance of shared political values, as we explained in the previous section. It must also appeal to evidence that is factually sound and based on valid methods of inquiry, such as those endorsed in the natural, health and social sciences.^7^

Hence, even if a public health measure such as the introduction of vaccine passports is justified based on a reasonable balance of shared political values, it may still not be publicly justified if it is grounded in flawed empirical evidence. For example, if vaccine passports were based on flawed or incomplete scientific premises, it would be illegitimate to enact them.^8^ But, equally, if and when opposition to vaccine passports is based on flawed scientific reasoning or pseudo-scientific views, it cannot provide legitimate grounds for rejecting their implementation. So what is the scientific evidence about vaccine passports?

First, it is necessary to assess whether and to what extent it is scientifically possible to establish that some individuals are immune to COVID-19, and more specifically the level and duration of their immunity. It is also important to stress that immunity may be acquired not only via COVID-19 vaccines but also by having contracted the virus (which is why vaccine passports, we have seen, are sometimes also referred to as ‘immunity’ passports). In both scenarios, it is important to establish the level and duration of immunity, including the level, duration and role of antibodies and T cells in our resistance to the virus (Moss 2022).

Second, it is necessary to evaluate whether and to what extent those considered immune are likely to transmit the virus to others. Since immunity is no guarantee of non-transmission—as we explained earlier, the vaccines available now are designed to make people less sick when they are exposed to the virus but not necessarily to stop people from becoming infected in the first instance—this is an important step.

Third, it is important to consider the evidence regarding the potential side effects of vaccine passports, especially negative side effects. We mentioned in the previous section that some oppose vaccine passports because they might impose a disproportionate burden on already vulnerable and marginalized groups. This is a moral claim (i.e., ‘it is wrong to exacerbate existing inequalities, including the marginalization and vulnerability of certain groups’) which presupposes some empirical evidence (i.e., ‘vaccine passports will or are likely to exacerbate existing inequalities, including the marginalization and vulnerability of certain groups’). Lest the moral claim lose force, that empirical evidence needs to be furnished and evaluated.^9^

## A Public Reason Ethics Framework for Vaccine Passports

Based on the foregoing analysis, we now present what we call a *public reason ethics framework* that can guide policymakers’ decisions about particular proposed vaccine passports. This framework , which is a variant of the framework developed by Barnhill and Bonotti (2022), outlines an analytic process—a process for analyzing the justifiability of proposed policies—grounded in the accessibility conception of public reason. This process consists of a number of questions that policymakers who have the power to implement vaccine passports should ask in order to identify the aims and potential unintended side effects of vaccine passports (including any evidence for them); assess whether and how these aims and side effects advance or encroach on political values; and evaluate whether vaccine passports can be justified based on a reasonable balance of political values. Use of this ethics framework can advance three goals. First, by using this framework and engaging in the analysis it outlines, policymakers (or other users) can assess the legitimacy of a proposed vaccine passport policy. Second, the process of considering potential side effects and downsides of policies may help policymakers to design more effective policies, given that side effects and downsides of policies may also be barriers to the policies’ effectiveness (for example, people may refuse to use a vaccine passport that threatens their privacy). Third, if the ethics framework is used not just by policymakers but also by citizens who provide input into policymaking, this is a way to render policymakers who design and plan to introduce a vaccine passport system more accountable to those citizens on whom the policy is imposed.

The framework’s first guiding question is the following:

(I) What are the public health-related goals of the proposed vaccine passport system?

In other words, this question asks policymakers to identify and explain what the direct and indirect public health purposes of the proposed system are. The potential public health goals of vaccine passports include: (1) reducing viral transmission in those places that require a vaccine passport for entry; (2) increasing vaccination rates in the community by attaching privileges to vaccination (or penalties to non-vaccination); (3) reducing overall viral transmission in the community via 1 and/or 2; and (4) reducing the rate of COVID-19 illness, hospitalization and death via 2 and 3. When designing a vaccine passport system, policymakers may have some of these goals in mind but not others; for example, the goal of a vaccine passport system might include 1 (reducing viral transmission in those places that require a passport for entry—though, as we have already explained, the vaccines on offer now do not seem to have been very effective at preventing transmission) but not 2 (increasing vaccination rates in the community).

After identifying the public health-related goals of the proposed vaccine passport system, policymakers are then asked to address two additional questions. First, (Ia) *How likely is it that the vaccine passport system will achieve its stated public health-related goals?* That is to say, what is the evidence for the public health-related effectiveness of the system? Answering these questions involves collecting any existing scientific evidence in support of vaccine passports; evaluating this evidence; discounting any evidence grounded in flawed data; and establishing whether there is scientific consensus regarding the non-flawed evidence and how it should be interpreted (also identifying majority and minority scientific positions, if relevant). Where there is insufficient scientific evidence, policymakers should consider funding new research, make and implement a provisional policy (while evidence is being gathered), and revise that policy periodically in the wake of the best evidence available. Second, (Ib), *Do the public health-related goals of the vaccine passport system promote shared political values and, if so, how?* For example, if it is effective at reducing the rate of COVID-19 illness, hospitalization and death, will the system thereby reduce public healthcare costs, thus promoting the common good?

The framework’s second guiding question is the following:

(II) Does the proposed vaccine passport system have other, non-public-health-related, goals?

As commentators have pointed out, if the public health goals of vaccine passports are achieved, this will also advance various other economic and social goals that are likely to promote the common good.^10^ For example, if vaccine passports are effective at reducing viral transmission in workplaces and schools that require vaccine passports, this may allow those places to reopen sooner and more safely, thereby enabling the earlier and safer resumption of work, commerce, and in-person schooling.

When addressing this second question, policymakers will also need to ask the following additional questions. First, (IIa) *How likely is it that the proposed vaccine passport system will achieve these other goals?* That is, what is the evidence (if any) for its effectiveness with regard to these other aims? Answering these questions will once again involve collecting relevant scientific evidence, evaluating it, and so on. And again, where there is insufficient scientific evidence, policymakers should consider funding new research, make and implement a provisional policy (while evidence is being gathered), and revise that policy periodically in the wake of the best evidence available. Second, (IIb) *Do these other goals of the vaccine passport system promote shared political values, and, if so, how?* For example, when people resume work, commerce and in-person schooling, this involves the restoration of freedom of movement (a central basic liberty) for them. The resumption of work and commerce will also help businesses to recover and will promote economic growth, which might increase equality of opportunity or promote justice in other ways. Third, (IIc) *Do these other goals encroach on shared political values and/or promote controversial non-political values and, if so, how?* For instance, is the aim of economic growth resulting from vaccine passports to favour specific economic actors (e.g., the businesses clamouring for workers to return to factories and offices, or the technology firms providing the infrastructure for digital passport systems) rather than the public more generally? If the justification for vaccine passports turns out to be based on non-political sectarian values and/or the partial interests of specific actors, policymakers should aim to either provide new public reasons for the policy or modify the policy (so that it can be justified by appealing to public reasons) or reject the policy.

The framework’s third guiding question is the following:

(III) Is the proposed vaccine passport system likely to have any unintended positive or negative side effects?

When addressing this third question, policymakers will also need to ask the following additional questions. First, (IIIa) *What are these side effects, and how likely are they?* The unintended positive and negative side effects of vaccine passports may be health- or non-health related. For example, would introducing vaccine passports open the door to requirements for ‘proof-of-status’ documents in other contexts? Would it unintentionally entrench the power of big tech firms?

Some side effects will be foreseeable and certain; for example, vaccine passports *will* make people’s freedom to access certain services dependent on being vaccinated and/or immune, thus limiting their freedom to some extent. And, some might argue, freedom is so central to liberalism that it should enjoy special consideration as the baseline of a liberal society, rather than its infringement being considered a mere ‘side effect’ of vaccine passports on a par with other side effects. However, while a presumption in favour of freedom is central to the liberal approach that underlies our framework, and while it is certainly true that vaccine passports infringe upon people’s freedom, so does becoming infected with COVID-19. Indeed, as Oberman points out,

[j]ust as lockdowns place people under threat of being fined for leaving home, so viruses place people under threat of infection. That threat prevents the conjunctive exercise of the freedom to perform actions that lead to infection and the freedom to perform actions that infection prevents […] What actions does infection prevent? The answer is clearest in the case of the symptomatic. Symptomatic people experience infirmity, limiting their movements. In the severest cases—coma or death—physical activity ceases altogether. It is worth pausing here to highlight that death itself, when due to other people and not purely natural causes (henceforth ‘unnatural death’), is a restriction of freedom. We risk missing this fact since standard examples of unfreedom—imprisonment, tyranny, and slavery—are suffered by people who go on living. Unnatural death restricts freedom by preventing victims from performing all the actions they could have performed during the rest of their natural lives. Indeed, unnatural death should arguably be our primary example of unfreedom. The victims of imprisonment, tyranny, and slavery can still perform some actions. The dead perform none (2022, p. 824).

Like lockdowns, vaccine passports infringe upon persons’ freedom but can also increase their freedom if they are effective in preventing infection. While the latter point, we explained earlier, is not self-evident—since current vaccines do not reduce to zero the risk of vaccinated people transmitting the virus—the more important point is that infringement of freedom is not a trump card that always tilts the balance in favour of less restrictive policies. Rather, the effects of a policy on freedom are one important dimension among others that should be considered.

To give another example of a side effect potentially resulting from a vaccine passport system, if the latter does not grant exemptions to people with religious opposition to vaccination—that is, people who are unvaccinated for religious reasons will still be denied entry to places that require vaccine passports—those people will be negatively affected. However, the severity of these negative effects will depend on which places require vaccine passports—e.g., many people would likely consider being prohibited from working or attending school in person worse than, say, being prohibited from eating indoors at a restaurant. Other side effects of a vaccine passport system might be uncertain—e.g., the system might serve to normalize privacy-invasive tracking of people’s movements in response to future public health problems, or it might not.

Having identified the potential positive and/or negative side effects of the proposed vaccine passport system, policymakers should then ask the following additional questions: (IIIb) *Do these side effects promote shared political values, and, if so, how?* And (IIIc) *Do these side effects encroach on shared political values and/or promote controversial non-political values and, if so, how?* For example, a vaccine passport that prevents people with religious objections to vaccination from returning to work encroaches on their labour rights as well as on their freedom to exercise one’s religion as one sees fit. Likewise, if certain groups of citizens lack equal access to vaccines and testing due to prior structural injustices, making these citizens’ freedom of movement dependent on vaccine passports is likely to perpetuate and compound those injustices, thus undermining the realization of shared political values such as equality of opportunity. When evidence suggests that a vaccine passport system is likely to impose disproportionate burdens on certain individuals or communities, policymakers should contemplate modifying or rejecting the policy.

The fourth and final guiding question is the following:

(IV) Does the proposed vaccine passport system strike a reasonable balance of political values?

In answering this question, policymakers need to ensure that all political values relevant to vaccine passports have been taken into account, and assess whether the balance between them is reasonable. For example, they should consider whether any specific political value has been ignored or assigned insufficient importance. If it turns out that this is the case, policymakers should consider rejecting the policy or modifying it so that the previously neglected political value(s) is (are) given more consideration. For example, if the vaccine passport system is initially implemented in order to restore freedom of movement and help restart economic activity, but without due consideration of privacy concerns or of the possibility that an enduring surveillance infrastructure is being created, these oversights could be remedied by imposing data minimization requirements on providers of vaccine passport systems and time limitations on their use. To give another example, it could be concluded that a vaccine passport system that does not exempt people with religious objections to vaccination fails to strike a reasonable balance between religious freedom and other values; in this case, the vaccine passport system could be modified to allow religious exemptions. Alternatively, the policymaker might conclude that even though the vaccine passport system encroaches on religious freedom as designed, this is a reasonable balance given the other political values that are at play.

## Conclusion

The ethical analysis of vaccine passports, like that of COVID-19 policies more generally, reveals ethical pros and cons, and the presence of disagreement among both policymakers and citizens. Therefore, liberal democratic governments require a decision-making process that grapples with that disagreement in the right way and helps public health policymakers to implement policies that are politically legitimate. In this paper, we have offered a framework, grounded in the accessibility conception of public reason, that can guide such policymakers through that decision-making process. This framework could also be used by citizens themselves, for example citizens participating in deliberative democracy forums and/or public consultation mechanisms. Using this framework to elicit structured public input could help policymakers to better understand potential side effects (especially negative ones) of a policy that were not obvious to them, to better recognize the range of ethical values and trade-offs at stake with public health measures, and to appreciate the disagreement about public health measures that exists among them and among citizens more generally. As Vallier points out,

Participatory democracies will focus on encouraging people to vote, under equal conditions, but will not expend great effort to increase the quality of deliberation in the choice of officials or policies. Such a democracy [i.e., deliberative democracy] will instead focus on directly and frequently consulting as many citizens as possible and ensuring that their input informs decisions…Deliberative democratic systems realize political equality through representative deliberation rather than mass participation. Participatory democracy is vulnerable to the fact that rich and highly educated citizens participate most often, and we typically do not want to compel voting and discussion in order to compensate. But deliberative systems can avoid this problem with microcosmic deliberation, where, again, persons are selected at random from the populace and then placed in face-to-face discussion. These individuals are given information about a policy choice and engage in structured deliberation; they are then given equal voting rights and vote on a proposed public policy (2019: 251; see also Vallier 2020 and Motchoulski 2020).

In response to some of the questions that we raised in the Introduction, we think that it is public health policymakers who should be tasked with crafting vaccine passport policy because they are best positioned to collect a broad range of information and data relevant to the policy. We also believe that public health policymakers should not simply follow their personal moral compass when making decisions about vaccine passports, or blindly defer to experts, or follow the will of the majority.^11^ Instead, by relying on a tool such as our ethics framework, they can engage in an analysis and decision-making process designed to yield a legitimate vaccine passport policy. Furthermore, by using this tool to obtain structured feedback from the public (for example, through deliberative forums or other mechanisms), policymakers can improve their accountability to the public.

While our analysis has focused on vaccine passports, the framework it exemplifies is portable, offering a useful guide for decision-making concerning other public health-related policies too. After all, most public health policy decisions are controversial and involve different and often conflicting values (as well as considerations about facts and evidence) that a public reason framework can help to organize and evaluate systematically. In fact, and for similar reasons, the framework could also be applied to policy areas that do not concern public health, including taxation and urban planning, for example. For those who, like us, believe that neither majority rule nor utilitarianism constitutes a suitable source of political legitimacy, the public reason framework that we have defended in this paper may represent a plausible alternative.

## Data Availability

Data sharing not applicable to this article as no datasets were generated or analysed during the current study.

## Footnotes

1 Technically, ‘vaccine passports’ certify that one has been vaccinated, while ‘immunity passports’ certify that one has immunity—i.e., the ability to resist an infectious disease—conferred either by vaccination or previous infection. In some contexts this distinction is important, because the different kinds of certifications create different incentives (e.g., by allowing different levels of freedom of movement), and thus raise subtly different ethical concerns (Phelan 2020). The focus of this paper, however, is not on the fine-grained aspects of these ethical considerations but rather on how to manage disagreements about them. As such, we use the terms interchangeably.

2 Some nasally administered vaccines might be able to do this, e.g., see Topol and Iwasaki (2022).

3 Though perhaps a false one, see World Health Organization 2021.

4 See also Bonotti (2020) for a criticism of Vallier’s (2019) attempt to respond to this challenge.

5 We say ‘may justify’ because Gaus’s common good requirement also demands that we consider other people’s rights and liberties, which mandatory vaccination may infringe upon. As Gaus explains, ‘we have…come to agree on some basic ingredients of a person’s good, such as protection of her bodily integrity, *basic liberties to make her decisions about most of the important aspects of her life*, control over basic resources needed to live a life, and so on. A rule that severely undermines a person’s ability to secure these elements of her good is surely not one that we can reasonably hold she has sufficient reasons to endorse’ (2010, p. 303, emphasis added). Therefore, whether the common good requirement justifies mandatory vaccination remains an open question.

6 Unlike Vallier’s, this position is more in line with our conceptualization of political legitimacy, in which the latter refers to the right of a political entity to pass and/or enforce a policy or law (Buchanan 2002)—a right which, in our view, is grounded in some practical social end captured by the idea of public reason. We acknowledge that a different understanding of political legitimacy, such as the view that a law or policy is legitimate when subjects have a duty to comply with it (Simmons 2001), may instead prevent this conclusion. More specifically, if persons are morally obligated to comply with laws or policies that they hold themselves to have conclusive (though unshared and inaccessible) reasons to reject, coercing them to obey those rules in such circumstances would fail to treat them as free and equal, for it would amount to subordinating their reasons and imposing upon them courses of conduct that they cannot help but regard as unreasonable. Simmons’s conceptualization of legitimacy may instead be more in line with an intelligibility conception of public reason, as the latter ensures that persons are only morally obligated to obey laws that they have conclusive reasons to endorse based on their conceptions of the good.

7 We assume that scientific standards of inquiry are widely endorsed in contemporary liberal democracies (see, e.g., Badano and Bonotti 2020).

8 Or, perhaps more pragmatically, public health policymakers may have to go ahead with and implement a provisional policy like vaccine passports in times of crisis like a pandemic while evidence is still being gathered. In such cases, they should revise that policy periodically in the wake of the best evidence available.

9 We recognize that while drug companies may be eager to fund research aimed at demonstrating the efficacy of vaccine passports, funding for research investigating the potential negative side effects of vaccine passports may be more limited. Given the limited space available, we set this issue aside here.

10 True, in certain contexts, such as the US, COVID-19 hospitalizations can be quite profitable for private healthcare institutions. However, it is not clear to what extent this may advance the common good as opposed to the mere private interest of such companies.

11 This is also because, as we explained earlier, sometimes public health policymakers may have to go ahead with and implement a provisional policy in times of crisis like a pandemic while evidence is still being gathered. In such cases, they should revise that policy periodically in the wake of the best evidence available, including that emerging from deliberative and consultation forums (as well as from the scientific community).
